# Editorial

**Published:** 1872-03-01

**Authors:** 


### MEDICAL EXAMINER.

*A Semi-Monthly Journal of Medical Sciences.*

EDITED BY

N. S. DAVIS, M. D., AND F. H. DAVIS, M. D.

Chicago, March 1st, 1872.

### EDITORIAL.

APOLOGY.—We had expected to get our issues up punctually to date by this time, but the junior editor rather unexpectedly left for a visit to Europe just after the issue of the fourth number of the present series, and the pressing duties connected with the last two weeks of the Medical College term has so far interfered with the work of the senior editor that the present number has fallen behind. The defect will soon be remedied.

COLLEGE COMMENCEMENT EXERCISES.—The Commencement exercises of the Chicago Medical College—Medical Department of the Northwestern University—took place on Monday and Tuesday, the 11th and 12th inst., in the College Hall, on the corner of Prairie avenue and 26th street. The exercises on Monday commenced at 2 o'clock p. m., and continued until 5 p. m., during which the whole graduating class were publicly examined, in addition to the ordinary examinations by the faculty during the previous week. Variety and interest was given to the occasion by the reading of theses by Wm. Butterfield, H. D. Porter and B. F. McMinnamy. On Tuesday the proper Commencement exercises began at 3 o'clock p. m.

The large lecture room was crowded with ladies and gentlemen, an excellent band of music was present to make melody, and Dr. E. O. Haven, President of the University, presided. After the opening prayer and music, the certificates of progress were distributed to the undergraduates of the junior and middle classes, followed by a few appropriate words of encouragement by the Dean of the Faculty.

The Professor of Practical and Clinical Surgery also gave special certificates to those candidates for graduation who had served as assistants in the surgical department of the hospital. The prize awarded for the best inaugural thesis, was taken by M. P. Hatfield; and that for the second best, by J. C. Patterson. Committee awarding the prizes also recommended for special mention the theses of H. D. Porter and F. B. E. Bockius. The degree of Doctor of Medicine was conferred on thirty-two regular students, followed by an appropriate charge by the President of the University, which was responded to on behalf of the graduating class, in an eloquent manner, by A. H. Levings. After music, the regular valedictory address was delivered by Prof. Thomas Bevan. We have not time or space to give an abstract of the address at present. It was a carefully prepared and eloquent production. The proceedings closed with music and the benediction.

The following are the names of the graduates:

*Ordinary Degrees*—J. M. Anderson, G. R. Bartron, John Bassian, S. L. Bedal, F. B. E. Bockius, C. W. Burrill, W. Butterfield, Henry Coakley, M. M. Grannis, H. C. Hastings, M. P. Hatfield, H. N. Hicks, A. E. Hoadley, J. O. Hobbs, A. H. Levings, Martin Matter, B. F. McMinnamy, C. S. McQuaid, F. C. Miller, A. K. Norton, J. C. Patterson, R. O. P. Phillips, Henry D. Porter, Nicholas Schilling, E. A. Shafer, A. H. Smith, S. S. Strayer, J. L. Twining, Joseph Van Buskirk, J. W. Waite, J. S. Wood, Henry Young.

*Ad eundem Degree*—Prof. T. Cleaver, Keokuk, Iowa.

